# Altered Iron Metabolism in Dogs with Naturally Occurring Cardiac Disease

**DOI:** 10.3390/biom16070997

**Published:** 2026-07-08

**Authors:** Carolina Frizzo-Ramos, Pavlos Doulidis, Ursula S. Kolm, Iwan A. Burgener, Franziska Roth-Walter, Nicole Luckschander-Zeller

**Affiliations:** 1 Small Animal Internal Medicine, Department for Companion Animals and Horses, University of Veterinary Medicine Vienna, 1210 Vienna, Austria; carolina.frizzo-ramos@vetmeduni.ac.at (C.F.-R.);; 2 Centre for Animal Nutrition and Welfare, University of Veterinary Medicine Vienna, 1210 Vienna, Austria

**Keywords:** inflammation, iron deficiency, nutritional immunity, veterinary cardiology

## Abstract

Cardiac disease represents a major cause of morbidity and mortality in dogs. In human cardiology, iron deficiency is a highly prevalent and clinically relevant comorbidity, contributing to exercise intolerance, symptom burden, and impaired quality of life independently of anemia, while correction with intravenous iron improves function and reduces hospitalization. Iron metabolism in dogs remains poorly characterized and typically considered only in the context of anemia. This study investigated iron metabolism and its regulatory pathways in dogs with cardiac disease, assessing whether alterations in iron handling and availability consistent with an iron-restricted phenotype are present. Hematologic indices, serum iron, total and unsaturated iron-binding capacity (TIBC, UIBC), ferritin, hepcidin, ceruloplasmin, C-reactive protein, and albumin were evaluated in 61 dogs comprising healthy controls, and dogs either with compensated cardiac disease (CCD group) or presenting acute decompensated congestive heart failure (ADCHF group). Dogs with cardiac disease exhibited evidence of systemic inflammation, reduced hematocrit, increased red blood cell distribution width, and decreased circulating iron. Both cardiac groups showed reduced TIBC and UIBC, suggesting decreased transferrin availability and reduced iron transport capacity, whereas ferritin concentrations did not differ between groups. Hepcidin concentrations were lower in CCD and not increased in ADCHF patients, suggesting complex and non-uniform regulation of iron homeostasis rather than a classic hepcidin-driven inflammatory pattern. In dogs with ADCHF, iron-binding capacity was independently associated with indices of cardiac remodeling, and additional correlation between inflammatory markers and iron-related parameters supported an interaction between inflammation, iron handling, and disease severity.

## 1. Introduction

Canine heart disease is a major cause of morbidity and mortality in adult and geriatric dogs, encompassing a range of chronic and progressive disorders that may result in exercise intolerance, respiratory compromise, reduced quality of life, and congestive heart failure (CHF). Myxomatous mitral valve disease (MMVD) is the most common acquired cardiac disease in dogs and a leading cause of CHF. Beyond hemodynamic dysfunction, CHF is increasingly recognized as a systemic condition involving inflammatory, metabolic, and neurohormonal alterations [1,2].

Chronic inflammation activates mechanisms of nutritional immunity, a host-defense strategy that restricts nutrient availability—particularly iron—through hepcidin-mediated inhibition of intestinal absorption and sequestration within macrophages [3,4,5]. While protective during acute infection, sustained activation of these mechanisms can impair host metabolism and tissue function.

In human medicine, heart failure represents a prototypical condition in which chronic low-grade inflammation persistently activates nutritional immunity, leading to iron deficiency (ID) that is frequently independent of anemia. Iron deficiency in human heart failure is associated with impaired exercise capacity, reduced quality of life, increased hospitalization rates, and adverse outcomes [6,7], and is an independent predictor of all-cause morbidity and mortality, irrespective of hemoglobin concentration [8,9]. Accordingly, iron deficiency—particularly functional iron deficiency (FID), characterized by preserved iron stores but reduced bioavailability—has become a therapeutic target in human heart failure [10,11,12].

Functional iron deficiency is thought to arise, at least in part, when inflammation-driven hepcidin upregulation limits intestinal iron absorption and systemic iron mobilization, although the relative contribution of these mechanisms in heart failure remains incompletely understood [3,4,5,13]. This explains the poor efficacy of oral iron therapy and the demonstrated benefits of intravenous iron supplementation in human heart failure [13,14,15,16,17]. These findings underscore the clinical relevance of identifying disease states in which nutritional immunity becomes chronically active.

In veterinary medicine, iron deficiency is traditionally considered only in the context of anemia, most commonly due to chronic blood loss and characterized by microcytic, hypochromic anemia [18]. Although hepcidin has been identified in dogs [19,20], nutritional immunity and functional iron deficiency are not established clinical concepts, and standardized criteria for diagnosing iron deficiency in non-anemic dogs are lacking. Nevertheless, subclinical iron deficiency has been reported in dogs exposed to chronic physiological or inflammatory stress [21,22].

Despite clear parallels between canine and human heart failure, iron metabolism in dogs with cardiac disease remains poorly characterized. Limited studies indicate lower serum iron concentrations in dogs with MMVD compared with healthy controls and a decline in iron parameters with disease progression [23,24], but these data do not distinguish between absolute and functional iron deficiency or address the role of hepcidin.

Critically, no study has comprehensively evaluated iron metabolism in dogs with cardiac disease within the framework of nutritional immunity, integrating circulating iron, iron-binding indices, ferritin, and hepcidin.

Therefore, the aim of this study was to characterize iron metabolism in dogs with cardiac disease. We hypothesized that chronic cardiac disease would be associated with inflammation-related alterations (nutritional immunity) in iron handling and reduced iron availability, potentially consistent with an iron-restricted phenotype independent of anemia, analogous to mechanisms described in human heart failure.

## 2. Materials and Methods

This was a combined retrospective and prospective observational study including three independent canine cohorts: (1) healthy controls, (2) dogs admitted to the Intensive Care Unit with acute decompensated congestive heart failure (ADCHF group), and (3) dogs with compensated cardiac disease (CCD group), managed under outpatient care by the cardiology service. Dogs with cardiac disease were grouped according to clinical status at the time of sampling into compensated cardiac disease (CCD) and acute decompensated congestive heart failure (ADCHF). CCD was defined as clinically stable cardiac disease without evidence of active congestive signs at the time of sampling, whereas ADCHF was defined as clinical heart failure requiring hospitalization, including cardiogenic pulmonary edema. This grouping was selected to reflect the physiological and clinical status at the time of blood sampling rather than the underlying cardiac diagnosis alone.

The samples from healthy dogs originated from an intervention study carried out by the first author in the Department of Dermatology of the Small Animal Hospital, University of Veterinary Medicine of Vienna, and all methods for obtaining healthy control samples were conducted in accordance with Austrian guidelines and regulations, with approval granted by the Ethics Committee of the University of Veterinary Medicine Vienna and the Austrian Federal Ministry of Science and Research (Ref: BMBWF 2023-0.618.811, approval date 13 July 2023).

For the healthy dogs, heparin-plasma samples were centrifuged at 3000 rpm for 3 min directly after collection and stored frozen at −20 °C until analysis. Animals over 1 year of age and 5 kg were considered after a diagnostic evaluation that included a thorough history, physical exam, complete blood count (CBC), and plasma biochemistry analysis. All blood samples were collected between 7:00 and 12:00, and all animals were fasting for at least 8 h before the blood collection. Dogs with CRP levels over 10 µg/L were considered to have subclinical low-grade inflammation and were excluded from the healthy control cohort.

For the ADCHF group, surplus plasma samples were prospectively collected from dogs admitted to the Intensive Care Unit of the Small Animal Clinic of the University of Veterinary Medicine Vienna over a period of six months. All samples consisted of residual material obtained during routine clinical procedures.

Dogs presenting with acute decompensated congestive heart failure were primarily admitted via the emergency service. As is typical in this clinical setting, a definitive cardiac diagnosis was not always available at the time of initial presentation. In such cases, a presumptive diagnosis of congestive heart failure was established based on the presence of dyspnea and evidence of cardiogenic pulmonary edema. Pulmonary edema was diagnosed based on interstitial or alveolar patterns on thoracic radiographs or, when radiography was not feasible due to patient instability, by the presence of B-lines on point-of-care ultrasonography in combination with subsequent clinical improvement following furosemide administration. Pulmonary hypertension, when present, was recorded as a concurrent echocardiographic finding and was not used as the primary criterion for inclusion in the ADCHF group.

For dogs without a previously established cardiac disease diagnosis at presentation, inclusion in the study required subsequent confirmation of the underlying cardiac disease by echocardiography performed by the attending cardiologist. Dogs without echocardiographic confirmation were excluded.

For dogs with MMVD, the last available American College of Veterinary Internal Medicine (ACVIM) stage assigned by the cardiology service was recorded descriptively based on available cardiology records. When no contemporaneous cardiology reassessment was available at the time of sampling, the authors did not retrospectively reassign ACVIM stage. For dogs with non-MMVD cardiac disease, the underlying cardiac diagnosis and clinical status at the time of sampling were recorded descriptively.

Exclusion criteria comprised hemorrhage (either active or anytime during 60 days prior to admission), presence of parasitic or hemoparasitic disease, bone marrow failure, hematologic neoplasia or cytotoxic chemotherapy. Dogs with advanced renal or hepatic failure were excluded where clinically identified. Chronic kidney disease (CKD) was staged according to the International Renal Interest Society (IRIS) guidelines for staging and treatment and considered severe at stages 3 or higher. Dogs with primary non-cardiogenic respiratory disease or pulmonary thromboembolism were excluded. These conditions were ruled out based on clinical assessment, thoracic imaging, echocardiography, and laboratory findings, including hematology and serum biochemistry. Additional diagnostics, such as D-dimer measurement, were performed when clinically indicated. Dogs were excluded when respiratory distress was better explained by a primary pulmonary disorder (e.g., infectious or inflammatory disease) or when findings were inconsistent with cardiogenic pulmonary edema.

Samples for the CCD group were obtained retrospectively and consisted of surplus material collected during routine clinical procedures. Inclusion criteria comprised dogs with diagnosed cardiac disease that were clinically stable and did not require hospitalization at the time of sampling. Due to the retrospective design and the requirement for available stored plasma samples, inclusion was limited to dogs for which residual material was still accessible. The CCD group was defined by compensated clinical status at the time of sampling and not by absence of ongoing cardiac medication or previous congestive episodes. All dogs underwent comprehensive cardiac assessment, including echocardiography, electrocardiography, and non-invasive blood pressure measurement, and were discharged on the same day following the appointment.

All dogs had complete blood cell counts performed, as well as biochemistry analysis. Blood smears were prepared and stained by an automated stainer using a modified Wright stain. Numerical changes in total leukocyte counts, neutrophils, and lymphocytes exceeding 25% of the upper or lower reference limit of the respective cell population and/or scatter plots indicating inappropriate cell separation prompted a microscopic slide evaluation by a senior technician to confirm abnormalities present in automatic counts, and if necessary, a microscopic count of 100 cells was carried out.

The measurement of canine ferritin was performed via a commercial ELISA essay according to manufacturer instructions (BlueGene E08F0136 Canine Ferritin Heavy Polypeptide ELISA, Shanghai, China) with samples diluted 1:4 for analysis. Hepcidin was measured with Duoset human hepcidin ELISA (due to the high cross-reactivity, R&D Systems DY8307, Minnneapolis, MN, USA) according to the manufacturers’ instructions and 1:50 sample dilution.

Ceruloplasmin activity was measurement with N, N-dimethyl-p-phenylenediamine dihydrochloride (DMPD, Sigma D4139, St. Louis, MO, USA), a compound that produces a long-lived radical cation, as described by Verde et al. 2002 [25]. To 100 µL 0.1 M acetate buffer, pH 4.8/well, 10 µL DMPD/well and 2 µL sample/standard (0–400 nM iron with 3% hydrogen peroxide) were added. Colorimetric reactions were monitored at 505 nm every 5 min for one hour. The oxidative activity was calculated based on the standard row and expressed in IU.

Serum iron, total iron-binding capacity (TIBC) and unsaturated iron-binding capacity (UIBC) were measured according to the method described by Yamashita et al. [26]. For serum iron measurements, plasma samples were diluted 1 + 1 in 0.89% NaCl, whereas for UIBC analysis, samples were 1 + 1 diluted with neutral 62 µmol/L ferrous solution in 75 mmol/L sodium hydrogen carbonate/375 mmol/L TRIS buffer, pH 8.4. Briefly, 15 µL of serum iron sample/UIBC sample/standards (for iron and copper) was mixed with 100 µL Nitro-Paps (Dojindo N031, Mashiki, Tabaru, Japan) color reagent (125 mM Nitropaps, 3% SDS, 45 mM ascorbic acid, 0.3 M acetate buffer pH 5), and absorbance was measured at 592 nm after 10 min to obtain serum iron and copper levels. Subsequently, 10 µL of 0.4 M thioglycolic acid in 50 mM sodium hydroxide was added (to remove copper from Nitropaps) and incubated for 5 min, and a second measurement was performed at 592 nm. The TIBC was calculated through summation of serum iron to UIBC as serum iron + UIBC = TIBC.

The statistical analysis was performed with GraphPad Prism 11.0.0 (84) (Graphpad Software LCC, 270 Park Avenue, NY, USA). Normality was tested with the Shapiro–Wilk test. Parametric data were then compared with Welch one-way ANOVA with Tukey post hoc, while the Kruskal–Wallis test with Dunn’s multiple comparisons test was used for not normally distributed data. Correlation analysis was performed with the Spearman rho test, followed by linear regression analysis where necessary. Exploratory multivariable linear regression analyses were performed to assess whether selected associations remained significant after adjustment for prespecified covariates. Statistical differences with *p* < 0.05 were considered significant.

Exploratory multivariable linear regression models were used as ANCOVA-like analyses to assess whether group effects for selected iron-related biomarkers persisted after adjustment for age, body weight, and sex. For each biomarker, disease group was included as a categorical predictor, while age, body weight, and sex were included as covariates. Because of the limited sample size and heterogeneous breed distribution, breed was not included as a covariate in the adjusted models.

To assess the potential influence of diagnostic heterogeneity, an exploratory sensitivity analysis was performed by repeating the main group comparisons after restricting the cardiac cohorts to dogs diagnosed with MMVD. This analysis was interpreted descriptively because of the reduced sample size and was used to assess whether the direction of the main findings was consistent with the full cohort.

## 3. Results

### 3.1. Characteristics of the Study Groups

Three study cohorts were compared, the first consisting of 22 surplus plasma samples of healthy dogs, comprising 16 females and 6 males. The breeds represented were Australian Shepard, Brabancon, Maltese, Labrador, Papillon, Poodle, Shetland Sheepdog, Collie, Chihuahua, Kannan, and Wolfdog, as well as mixed-breed dogs. Median age was 7 years old (range 1–11).

The ADCHF group consisted of 25 dogs, of which 12 were male and 13 were female. Chihuahua was the most common breed (48%, *n* = 12), followed by Yorkshire Terrier, Maltese, Shih Tzu, Cavalier King Charles Spaniel, Magyar Vizsla, Labrador Retriever, Bichon Frisé, Irish Wolfhound, Shetland Sheepdog, and mixed-breed dogs. The median age was 12 years (range, 4–15 years). Based on the cardiology records, MMVD was recorded in 19 dogs and DCM in one dog. Pulmonary hypertension was documented in six dogs and was reported descriptively as an echocardiographic finding rather than as the primary mechanistic explanation for pulmonary edema. Dogs were assigned to the ADCHF group based on clinical decompensation with cardiogenic pulmonary edema requiring hospitalization at the time of sampling. In cases in which pulmonary hypertension was recorded without a definitive left-sided primary diagnosis assigned by the cardiologist, no retrospective MMVD diagnosis was attributed by the authors. Where ACVIM stage is reported, it reflects the last available cardiology-recorded stage and not necessarily restaging at the time of ADCHF presentation.

Clinical and hematological parameters from all groups, including treatment status and selected echocardiographic parameters, were retrieved from medical records where available and are summarized descriptively in Table 1. The CCD group consisted of 14 outpatient dogs with cardiac disease that were clinically stable at the time of sampling, of which 4 were female and 10 were male. No breed was overrepresented in this group, and the participating breeds were Pinscher, Boxer, Bull Terrier, Doberman Pinscher, Poodle, Chihuahua, and mixed-breed dogs. The median age was 14 years (range, 8–18 years). Twelve dogs were diagnosed with MMVD, and based on the last available cardiology-recorded ACVIM stage, these were described as stage B2 (*n* = 7) or stage B1 (*n* = 5). One dog was diagnosed with pulmonary hypertension, and one dog had dilated cardiomyopathy. Four dogs in the CCD group had diuretics listed among their ongoing cardiac medications; however, the indication for diuretic treatment could not be retrospectively confirmed in all cases. Because ACVIM stage reflected the last available cardiology-recorded stage rather than standardized restaging at the time of sampling, these data were reported descriptively. These dogs were retained in the CCD group because grouping was based on clinical status at blood collection: they were clinically stable, were managed as outpatients, did not require hospitalization, and had no evidence of active congestive signs at that visit.

**Figure 1:**
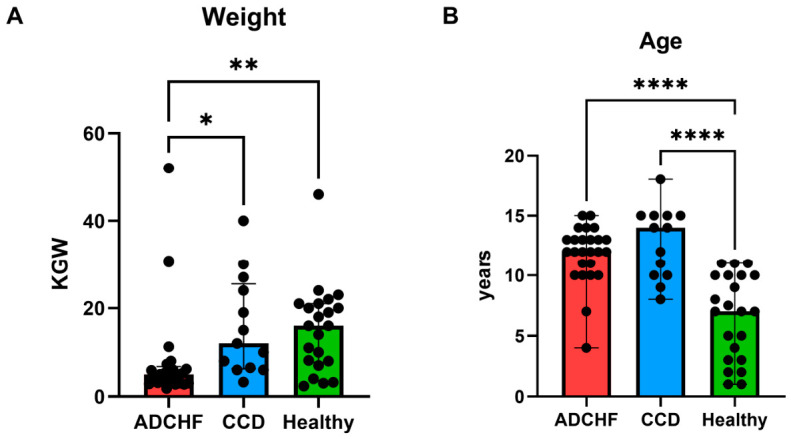
(**A**) Scatter dot plot (median with range) showing the significant difference in weight between the acute decompensated congestive heart failure (ADCHF) group and the compensated cardiac disease (CCD) group and the ADCHF group and the healthy dogs. (**B**) Scatter dot plot (median with range) showing the significant difference in age between the ADCHF group and the CCD group when compared to healthy dogs. Asterisks indicate statistically significant pairwise comparisons: * *p* < 0.05; ** *p* < 0.01; **** *p* < 0.0001.

Patients in both the ADCHF and CCD groups were significantly older than healthy dogs (pairwise comparisons *p* < 0.001), but no difference in age was detected between ADCHF and CCD patients (Figure 1). Sex distribution varied numerically between groups, with females predominating in the healthy control group, an approximately balanced sex distribution in the ADCHF group, and males predominating in the CCD group. Breed distribution was heterogeneous, with overrepresentation of small-breed dogs, particularly Chihuahuas, in the ADCHF group. Therefore, sex was included as a covariate in adjusted analyses, while breed was summarized descriptively but not included in multivariable models because of sparse breed categories.

In an exploratory MMVD-only sensitivity analysis, the direction of the main findings was consistent with the full cohort, including lower serum iron concentrations in ADCHF dogs compared with both CCD and healthy dogs, lower TIBC in both cardiac groups compared with healthy dogs, and lower UIBC in CCD dogs compared with healthy dogs. Because the study was designed to evaluate naturally occurring cardiac disease according to clinical status at sampling, the full cohort was retained as the primary analysis.

### 3.2. Lower Hematocrit in Heart Failure Patients

**Figure 2:**
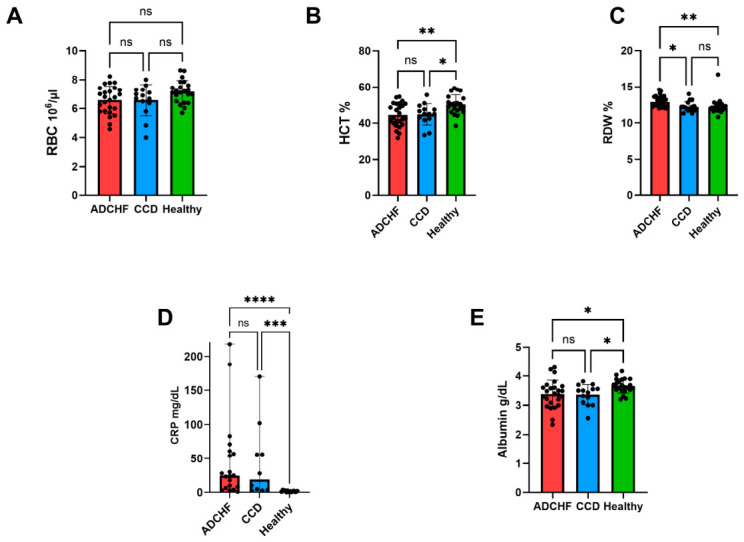
Scatter dot plot of selected relevant routine hematological parameters, with pairwise comparisons of the three groups. (**A**) No significant difference in the red blood cell (RBC) count between the three groups. (**B**) Dogs with acute decompensated congestive heart failure (ADCHF) have significantly lower hematocrit (HCT) compared to healthy dogs (*p* = 0.0047), while dogs in the compensated cardiac disease (CCD) group have lower hematocrit compared to healthy dogs (*p* = 0.0196). (**C**) Dogs in the ADCHF group have higher red cell distribution width (RDW) compared with healthy dogs (*p* = 0.0011) and dogs in the CCD group (*p* = 0.0153). (**D**) C-reactive protein (CRP) was significantly higher in the ADCHF group compared to healthy controls and in the CCD group compared to healthy dogs (*p* = 0.001). (**E**) Serum albumin (ALB) levels were significantly lower in the ADCHF compared to healthy dogs (*p* = 0.0417) and lower in the CCD group (*p* = 0.045), whereas no significant differences were detected between the CCD and ADCHF groups. Asterisks indicate statistically significant pairwise comparisons: * *p* < 0.05; ** *p* < 0.01; *** *p* < 0.001; **** *p* < 0.0001. Non-significant comparisons are indicated as *ns.*

In the first step, we assessed hematological parameters to evaluate alterations in erythrocyte indices and potential subclinical nutritional or metabolic effects associated with cardiac disease progression. Hematological parameters are summarized in Figure 2. Red blood cell count did not differ significantly between the three groups (Figure 2A). In contrast, hematocrit (HCT) was significantly lower in both ADCHF dogs (mean 44.5%, SD ± 6.45%) and CCD dogs (mean 45.0%, SD ± 5.90%) compared with healthy controls (mean 50.35%, SD ± 5.35%; *p* = 0.0047 and *p* = 0.0196, respectively), whereas no difference was observed between the two cardiac groups (Figure 2B).

Red cell distribution width (RDW) was significantly higher in the ADCHF group (median 12.90%, range 12–14.60%) compared with healthy dogs (median 12.05%, range 10.80–16.70%) (*p* = 0.0011) and dogs in the CCD group (median 12.2%, range 11.3–14%) (*p* = 0.0153) (Figure 2C). Overall, dogs with cardiac disease had lower hematocrit than healthy dogs, while increased RDW was most evident in the ADCHF group.

### 3.3. Inflammation in Patients with Heart Disease

The concentration of C-reactive protein (CRP) was significantly higher in the ADCHF group (median 24.50 mg/L, range 1–218.0 mg/L) compared to healthy controls (median 1.3 mg/L, range 0.1–3.8 mg/L) (*p* < 0.0001) and in the CCD group (median 19.0 mg/L, range 2.6–170 mg/L) compared to healthy dogs (*p* = 0.001), while no significant difference was observed between the ADCHF and CCD groups (Figure 2D). Serum albumin levels were significantly lower in the ADCHF group (mean 3.38 g/dL, SD ± 0.47 g/dL) compared to healthy dogs (mean 3.66 g/dL, SD ± 0.24 g/dL) (*p* = 0.0417) and lower in the CCD group (mean 3.36 g/dL, SD ± 0.34 g/dL) (*p* = 0.045), whereas no significant differences were detected between the CCD and ADCHF groups (Figure 2E).

### 3.4. Inflammation-Related Biomarkers of Iron Metabolism in Canine Cardiac Disease

**Figure 3:**
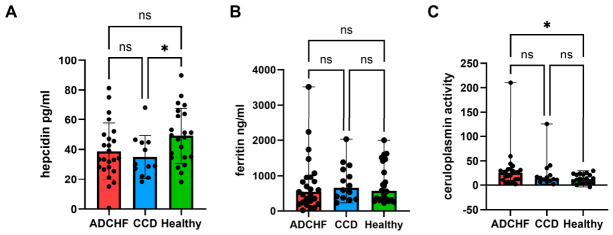
Scatter dot plot with pairwise comparisons between the 3 groups. (**A**) Comparison of hepcidin levels. Dogs in the compensated cardiac disease (CCD) group show significantly lower hepcidin levels compared to healthy dogs (*p* = 0.0316). No significant differences were detected between healthy dogs and the acute decompensated congestive heart failure (ADCHF) group or between the ADCHF and CCD groups, although this difference was close to significance. (**B**) Comparison of ferritin levels. Ferritin levels did not differ significantly between healthy dogs, dogs with ADCHF, and CCD patients, with substantial variability observed within all groups. (**C**) Dogs in the ADCHF group had higher ceruloplasmin activity compared with healthy dogs (*p* = 0.0189). No significant difference was detected between dogs with ADCHF and the CCD group, and between the CCD group and healthy dogs. Asterisks indicate statistically significant pairwise comparisons: * *p* < 0.05; Non-significant comparisons are indicated as *ns*.

Next, we assessed plasma hepcidin, ferritin, and ceruloplasmin to evaluate inflammatory regulation of iron metabolism, iron storage, and iron mobilization in dogs with cardiac disease. Plasma hepcidin concentrations differed significantly between healthy dogs (mean 49.21 pg/mL, SD ± 18.54 pg/mL) and dogs in the CCD group (mean 35 pg/mL, SD ± 14.28 pg/mL) (*p* = 0.0316), with lower concentrations observed in the CCD dogs (Figure 3A). No significant differences in hepcidin concentrations were detected between healthy dogs and the ADCHF group or between the ADCHF and CCD groups.

Ferritin levels did not differ significantly between healthy dogs, ADCHF dogs, and CCD patients, with substantial variability observed within all groups (Figure 3B).

Ceruloplasmin is a ferroxidase involved in iron metabolism, facilitating the oxidation of ferrous to ferric iron and thereby enabling its binding to transferrin and mobilization into circulation. Plasma ceruloplasmin ferroxidase activity was significantly higher in dogs with ADCHF (median 26.35, range 2–210) compared with healthy dogs (median 13.19, range −3.5–30.20) (*p* = 0.0189) (Figure 3C). No significant difference was detected between dogs with ADCHF and the CCD group, and between the CCD group and healthy dogs.

In summary, hepcidin was lower in CCD dogs compared with healthy dogs, ferritin did not differ significantly between groups, and ceruloplasmin activity was higher in ADCHF dogs compared with healthy dogs.

### 3.5. Reduction in Iron Availability with Advancing Cardiac Disease

We assessed serum iron and iron-binding indices (TIBC and UIBC) to evaluate iron availability and distinguish between absolute and functional iron deficiency across disease stages.

**Figure 4:**
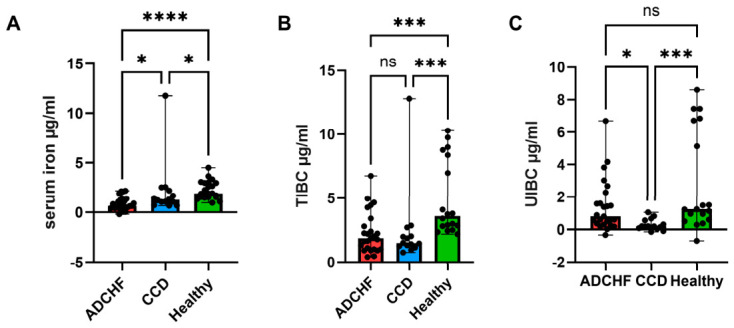
Scatter dot plot with pairwise comparisons between the 3 groups. (**A**) Comparison of serum iron levels. Dogs in the acute decompensated congestive heart failure (ADCHF) group had significantly lower serum iron levels compared to healthy dogs (*p* < 0.0001) and compensated cardiac disease (CCD) patients (*p* = 0.0225). Dogs in the CCD group exhibited significantly lower serum iron concentrations compared with healthy dogs (*p* = 0.044). (**B**) Comparison of total iron-binding capacity (TIBC). TIBC was significantly higher in healthy dogs compared with both dogs with ADCHF (*p* = 0.0006) and CCD (*p* = 0.0007), while no significant difference was observed between the two disease groups. (**C**) Comparison of unsaturated iron-binding capacity (UIBC). UIBC differed between groups, with significantly higher values in healthy dogs compared with CCD patients (*p* = 0.0005) and higher values in dogs with ADCHF compared with CCD patients (*p* = 0.0137), whereas no significant difference was detected between healthy dogs and dogs with ADCHF. Asterisks indicate statistically significant pairwise comparisons: * *p* < 0.05; *** *p* < 0.001; **** *p* < 0.0001. Non-significant comparisons are indicated as *ns.*

Serum iron concentrations differed significantly among groups (Figure 4A). Dogs with ADCHF exhibited markedly lower serum iron levels (median 0.64 µg/mL, range −0.19–2.16 µg/mL) compared with both healthy dogs (median 1.89 µg/mL, range 0.95–4.50 µg/mL; *p* < 0.0001) and CCD dogs (median 1.26 µg/mL, range 0.65–11.75 µg/mL; *p* = 0.0225). Serum iron concentrations were also significantly lower in CCD dogs compared with healthy controls (*p* = 0.044).

Total iron-binding capacity (TIBC) was significantly higher in healthy dogs (median 3.575 µg/mL, range 2.18–10.29 µg/mL) than in both dogs with ADCHF (median 1.85 µg/mL, range 0.40–6.74 µg/mL; *p* = 0.0006) and CCD (median 1.48 µg/mL, range 0.75–12.81 µg/mL; *p* = 0.0007), with no significant difference between the two disease groups (Figure 4B).

Unsaturated iron-binding capacity (UIBC) also differed between groups (Figure 4C). Healthy dogs showed significantly higher UIBC values (median 1.24 µg/mL, range −0.70–8.60 µg/mL) compared with CCD dogs (median 0.19 µg/mL, range −0.15–1.05 µg/mL; *p* = 0.0005). Dogs with ADCHF had higher UIBC values (median 0.80 µg/mL, range −0.34–6.70 µg/mL) than CCD dogs (*p* = 0.0137), whereas no significant difference was detected between healthy dogs and dogs with ADCHF.

Together, these findings demonstrate lower circulating iron concentrations in dogs with cardiac disease, with the most pronounced reduction observed in dogs with ADCHF. Iron-binding capacity was also reduced in dogs with cardiac disease, although this change did not follow the same progressive pattern as serum iron.

### 3.6. Adjusted Analyses of Iron-Related Biomarkers

To assess whether group differences in iron-related biomarkers persisted after adjustment for demographic variables, exploratory multivariable linear regression models were performed using disease group as a categorical predictor and age, body weight, and sex as covariates. Disease group remained significantly associated with serum iron concentrations after adjustment for these covariates (F_2_,_54_ = 4.852, *p* = 0.0115), whereas body weight, age, and sex were not significantly associated with serum iron. Compared with dogs with ADCHF, adjusted serum iron concentrations were significantly higher in both CCD dogs (*p* = 0.0272) and healthy dogs (*p* = 0.0161).

In contrast, disease group was not significantly associated with TIBC, ferritin, hepcidin, or ceruloplasmin after adjustment for age, body weight, and sex. None of the demographic covariates were significantly associated with TIBC, ferritin, hepcidin, or ceruloplasmin. Together, these adjusted analyses indicate that reduced serum iron was the most robust iron-related alteration in this cohort, whereas group differences in other iron-related biomarkers were more variable after demographic adjustment.

### 3.7. Association Between Iron Metabolism, Inflammation, and Cardiac Remodeling

To further explore the biological relevance of iron alterations, associations between iron-related parameters, inflammatory markers, and echocardiographic indices were evaluated.

**Figure 5:**
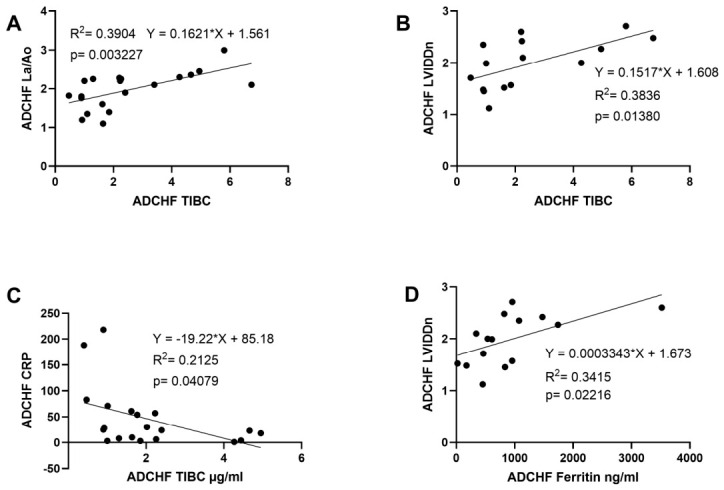
Associations between iron-related parameters, inflammatory markers, and echocardiographic indices in dogs with acute decompensated congestive heart failure (ADCHF). The lines represent linear regression fits. Correlation coefficients (r) and *p*-values are indicated within each panel. All analyses were performed in dogs with ADCHF. (**A**) Scatter plot showing the relationship between total iron-binding capacity (TIBC) and left-atrial-to-aortic ratio (La/Ao). (**B**) Scatter plot showing the relationship between TIBC and normalized left ventricular internal diameter in diastole (LVIDdn). (**C**) Scatter plot showing the relationship between ferritin and LVIDdn. (**D**) Scatter plot showing the relationship between C-reactive protein (CRP) and TIBC.

For the ADCHF group, TIBC showed a moderate positive correlation with both indices of cardiac remodeling, including La/Ao (r = 0.64, *p* = 0.003) and LVIDdn (r = 0.62, *p* = 0.015). Linear regression analysis confirmed a significant association between TIBC and La/Ao (R^2^ = 0.39, β = 0.162, *p* = 0.003) (Figure 5A), as well as between TIBC and LVIDdn (R^2^ = 0.27, β = 0.143, *p* = 0.032) (Figure 5B). In addition, ferritin was positively correlated with LVIDdn (r = 0.65, *p* = 0.010), whereas CRP was inversely correlated with TIBC (r = −0.55, *p* = 0.011). Serum iron and hepcidin were not significantly associated with echocardiographic parameters (all *p* > 0.05), and albumin showed a borderline association with LVIDdn (r = 0.51, *p* = 0.054). As expected, a strong positive correlation was observed between La/Ao and LVIDdn (r = 0.75, *p* = 0.002).

In multivariable regression analysis including TIBC, albumin, and ferritin, TIBC remained independently associated with both La/Ao and LVIDdn, whereas albumin and ferritin were not significant predictors.

In contrast, for the CCD group, no significant associations were observed between TIBC and echocardiographic parameters (LVIDdn: r = 0.02, *p* = 0.951; La/Ao: r = 0.27, *p* = 0.394). Ferritin and CRP were likewise not significantly correlated with cardiac dimensions (all *p* > 0.05). A moderate correlation between La/Ao and LVIDdn was observed in both groups (inpatients: r = 0.75, *p* = 0.002; outpatients: r = 0.63, *p* = 0.040).

These associations were observed in the ADCHF group but not in the CCD group.

## 4. Discussion

In the present study, dogs with cardiac diseases—particularly those with CHF—exhibited evidence of hematologic, inflammatory and iron-related alterations that together indicate a disruption of iron homeostasis in the context of chronic inflammation. Similar to observations in human heart failure, these changes were evident in the absence of overt anemia and suggest early metabolic and nutritional consequences of cardiac disease progression [27].

### 4.1. Early Hematologic Alterations and Impaired Erythropoiesis

Dogs with cardiac disease showed reduced hematocrit without a concomitant reduction in red blood cell count, indicating that overt anemia was not yet established. In dogs with congestive heart failure, lower hematocrit may partly reflect altered fluid balance or hemodilution, particularly in the context of fluid retention and diuretic treatment. Accordingly, hematocrit values in dogs with cardiac disease should be interpreted with caution, as changes may not solely reflect erythropoietic status but also shifts in plasma volume associated with disease severity and treatment. However, the concomitant increase in RDW, especially in dogs with ADCHF, suggests that the hematologic changes are unlikely to be explained by dilution alone.

Increased RDW reflects greater erythrocyte size heterogeneity and is commonly associated with impaired erythrocyte maturation, nutritional deficiencies including iron, vitamin B12, and folate, and inflammation-related iron restriction. Findings regarding RDW in canine cardiac disease are variable: while several studies have reported increased RDW or associations with disease severity and erythrocyte indices in dogs with MMVD, other reports indicate that RDW may remain within reference limits or not differ significantly between groups [28,29]. This variability may reflect differences in disease stage, population characteristics, or methodological factors, and suggests that RDW should be interpreted in conjunction with other hematologic and biochemical markers. In the present cohort, the combined pattern of lower hematocrit and increased RDW is therefore more consistent with early erythropoietic stress or iron-restricted red cell production than with a purely dilutional effect.

### 4.2. Systemic Inflammation, Acute-Phase Response and Protein Redistribution

Clear evidence of systemic inflammation was present in dogs with cardiac disease. Both ADCHF and CCD patients exhibited significantly elevated CRP concentrations, accompanied by significantly reduced serum albumin levels. Albumin is a negative acute-phase protein and a major indicator of circulating protein reserves; its reduction reflects inflammation-driven reprioritization of hepatic protein synthesis and altered nutrient allocation rather than simple dietary insufficiency. This pattern has been described in human heart failure and is associated with adverse metabolic and clinical outcomes [30,31]. The more pronounced inflammatory changes observed in dogs with ADCHF indicate that inflammatory burden increases with disease severity, while the presence of similar but milder alterations in CCD dogs suggests that these processes begin early in the disease course.

While clinical manifestations such as effusions may influence serum albumin concentrations in dogs with congestive heart failure, mild ascites were only observed in three patients in the present study population and pleural effusion was only present in one subject. These clinical characteristics of the study population suggest that fluid-related losses are unlikely to be a major driver of the observed hypoalbuminemia. Accordingly, the decrease in albumin more likely reflects inflammation-associated changes in hepatic protein allocation and acute-phase responses, although multifactorial contributions cannot be fully excluded.

In addition to hypoalbuminemia, dogs with CHF exhibited significantly elevated ceruloplasmin ferroxidase activity. Ceruloplasmin is a positive acute-phase protein but also plays an important role in iron metabolism through its ferroxidase activity, which facilitates the oxidation of ferrous (Fe^2+^) to ferric (Fe^3+^) iron, enabling its binding to transferrin and export into circulation. Increased ceruloplasmin levels therefore likely reflect both heightened inflammatory activity and a compensatory attempt to mobilize intracellular iron stores in response to reduced circulating iron availability.

It should be noted, however, that the assay used in this study measures ceruloplasmin ferroxidase activity rather than total ceruloplasmin protein concentration, and therefore does not fully capture all aspects of ceruloplasmin function in vivo, including its role as an acute-phase reactant or its interactions with cellular iron transport pathways. Accordingly, the observed increase in ceruloplasmin activity should be interpreted as indicative of altered iron-related enzymatic activity rather than a comprehensive assessment of ceruloplasmin biology.

### 4.3. Functional Iron Deficiency and Impaired Iron Transport

Consistent with this inflammatory profile, serum iron concentrations were significantly reduced in dogs with cardiac disease, with the most pronounced reductions observed in ADCHF patients and intermediate reductions in CCD dogs. Importantly, TIBC was also reduced in both cardiac groups, indicating decreased transferrin availability. This pattern contrasts with absolute iron deficiency in non-inflammatory states, where transferrin synthesis and TIBC are typically increased [32].

Instead, reduced TIBC in this context may reflect inflammation-associated changes in hepatic protein allocation and transferrin availability, rather than direct evidence of reduced hepatic protein synthesis, and may additionally be influenced by nutritional or metabolic alterations associated with cardiac disease. The concurrent decrease in albumin, a negative acute-phase protein synthesized by the liver, provides indirect support for inflammation-associated shifts in hepatic protein production, although direct assessment of hepatic synthetic function was beyond the scope of this study. Together, the combination of low serum iron and reduced iron-binding capacity supports a pattern consistent with functional iron deficiency, characterized by impaired iron bioavailability despite preserved or masked iron stores [33].

The persistence of the serum iron group effect after adjustment for age, body weight, and sex supports reduced circulating iron as the most robust iron-related alteration in this cohort. In contrast, adjusted analyses did not confirm independent group effects for TIBC, ferritin, hepcidin, or ceruloplasmin. Because age, body weight, and sex were not significantly associated with these biomarkers, the loss of significance after adjustment may reflect reduced statistical power, biological variability, group heterogeneity, or additional unmeasured clinical factors rather than a single dominant demographic effect. These markers may therefore still provide biologically relevant information, but their interpretation appears more variable and context-dependent than serum iron.

### 4.4. Ferritin and Hepcidin Reflect Competing Regulatory Signals

Classical markers of iron storage and regulation behaved in a manner consistent with complex, competing regulatory influences. Ferritin concentrations did not differ between groups, despite lower circulating iron and clear evidence of systemic inflammation in cardiac patients. As ferritin acts both as the principal iron-storage protein and as an acute-phase reaction marker, inflammation-driven increase may obscure underlying iron depletion, limiting its utility as a standalone indicator of iron status in chronically inflamed individuals [34].

Similarly, serum hepcidin concentrations were not increased in dogs with ADCHF and were significantly lower in CCD patients compared with healthy controls. While inflammation is classically associated with hepcidin upregulation and iron sequestration, hepcidin is suppressed in states of iron deficiency and increased erythropoietic demand [35]. When iron deficiency and inflammation coexist, hepcidin expression reflects the balance between opposing regulatory signals, and may remain inappropriately normal or reduced when erythropoietic drive counteracts inflammation-induced hepcidin upregulation [36]. The lower hepcidin levels observed in CCD dogs, in the context of reduced circulating iron, may represent a compensatory response aimed at enhancing iron absorption and mobilization. In dogs with ADCHF, the absence of hepcidin upregulation despite pronounced inflammation suggests a more complex regulatory environment in which inflammatory signaling and erythropoietic drive counterbalance each other, resulting in hepcidin concentrations that appear deceptively similar to those of healthy dogs. Comparable dissociations between inflammation, iron status, and hepcidin regulation have been reported in chronic disease states in humans [37,38].

Importantly, circulating biomarkers provide only an indirect representation of iron homeostasis and may not fully reflect tissue-level iron distribution or cellular iron availability. Therefore, while the observed changes are consistent with altered iron handling, they do not allow direct assessment of iron sequestration within specific compartments or its functional impact on erythropoiesis [39].

### 4.5. Inflammation-Associated Alterations in Iron and Protein Handling in Canine Cardiac Disease

Taken together, the constellation of reduced serum iron, decreased iron-binding capacity, elevated inflammatory markers, increased ceruloplasmin, reduced albumin, and altered erythrocyte indices indicates a coordinated inflammatory response that alters nutrient handling and redistribution in dogs with cardiac disease. Rather than reflecting isolated deficiencies, these changes are consistent with processes described within the framework of nutritional immunity, in which iron and protein resources are actively sequestered or reprioritized during sustained inflammation. While adaptive in acute infection, prolonged activation of these mechanisms may lead to functional nutritional deficits that impair erythropoiesis and tissue metabolism, even in the absence of overt anemia or clinically apparent malnutrition.

A plausible contributing pathway for this phenotype is inflammation-associated iron restriction, in which acute-phase responses reduce circulating iron availability through decreased transferrin synthesis, altered iron mobilization, and competing regulation of hepcidin by inflammatory and erythropoietic signals. However, these mechanisms were not directly assessed in the present study, and the observed alterations should therefore be interpreted as compatible with, but not definitive proof of, functional iron deficiency or chronic activation of nutritional immunity.

### 4.6. Dysregulation of Iron Homeostasis in Acute Decompensated Congestive Heart Failure

In the present study, iron-related parameters were not only altered in dogs with CHF but also associated with indices of cardiac remodeling in a stage-dependent manner. In hospitalized dogs with ADCHF, TIBC, reflecting iron transport capacity, was independently associated with both La/Ao and LVIDdn, suggesting a link between iron handling and the severity of cardiac remodeling.

Importantly, although TIBC was reduced overall in dogs with cardiac disease compared with healthy controls, exploratory analyses within the ADCHF group demonstrated that, among already decompensated dogs, relatively higher TIBC values were associated with greater cardiac remodeling. This finding indicates that regulation of iron transport capacity in advanced disease is not unidirectional but likely reflects the net effect of competing physiological processes rather than inflammation alone.

Under inflammatory conditions, TIBC would typically be expected to decrease due to reduced hepatic transferrin production as part of the negative acute response.

This pattern was observed at the group level in the present study, where both ADCHF and CCD dogs exhibited lower TIBC compared with healthy controls. However, within the ADCHF group, the positive association between TIBC and indices of cardiac remodeling suggests that, in more advanced stages of disease, additional regulatory mechanisms may counteract or override inflammation-associated suppression of transferrin.

One plausible explanation is the presence of increased iron demand associated with stimulated erythropoiesis or tissue hypoxia in advanced heart failure. In this context, upregulation of transferrin synthesis and iron transport capacity may occur as a compensatory response maintaining iron delivery to metabolically active tissues, including the bone marrow. Such a response could result in relatively higher TIBC values in dogs with more pronounced cardiac remodeling, despite the presence of systemic inflammation.

Furthermore, these patterns occurred in the context of non-elevated or even reduced hepcidin concentrations despite evidence of systemic inflammation, further supporting the notion that iron regulation in this setting may not be driven by inflammation alone.

Hepcidin expression is regulated by opposing signals, including inflammatory cytokines, iron availability, and erythropoietic activity. In states where iron deficiency or increased erythropoietic drive coexists with inflammation, hepcidin levels may remain inappropriately normal or suppressed, thereby permitting continued iron mobilization despite inflammatory signaling.

Taken together, these findings support a model of dysregulated iron homeostasis in advanced cardiac disease, in which inflammation, iron demand, and erythropoietic signaling interact to shape iron availability and transport. Rather than reflecting a classical inflammation-mediated iron sequestration phenotype, the observed pattern is more consistent with an iron-restricted state in which iron supply is insufficient relative to physiological demand.

Importantly, these associations were not observed in dogs who had CCD, suggesting that the interaction between iron metabolism and cardiac remodeling becomes more apparent in the decompensated stage of disease.

This stage-dependent relationship supports the concept that alterations in iron handling may evolve over the course of disease progression, becoming more pronounced and functionally relevant in advanced heart failure.

These observations are in line with findings in human heart failure, where complex interactions between inflammation, iron metabolism, and cardiac function have been described, and where iron deficiency—whether absolute or functional—has been associated with disease severity and adverse outcomes.

While the present study does not allow definitive mechanistic conclusions, the identified associations provide clinically grounded and biologically plausible evidence linking iron metabolism to cardiac remodeling in dogs with CHF and support the need for further mechanistic studies and longitudinal investigations.

### 4.7. Limitations

The study design included both retrospective and prospective data, which may have introduced variability in data completeness and standardization. Furthermore, measurements were obtained at a single time point, precluding assessment of temporal relationships between inflammation, iron metabolism, and erythropoietic changes. As such, causality cannot be inferred, and the observed alterations may represent either primary mechanisms or secondary adaptations to cardiac disease.

Additionally, circulating biomarkers were used to assess iron status and inflammatory responses. While these markers provide valuable clinical information, they represent indirect measures and may not fully reflect tissue-level iron distribution or cellular iron availability. Direct assessment of iron stores, hepcidin dynamics, or bone marrow iron utilization was beyond the scope of this study.

It should be noted that the study population included dogs with heterogeneous cardiac diseases. While ACVIM staging was recorded descriptively for patients with MMVD, dogs with other cardiac conditions were described according to the underlying diagnosis, as ACVIM staging is not universally applicable. In addition, not all dogs underwent repeat standardized cardiologic staging at the time of sampling. Consequently, reported ACVIM stage reflects the most recent available cardiology assessment and should not necessarily be interpreted as contemporaneous restaging at the time of blood collection. This limitation should be considered when interpreting group comparisons.

Although age, body weight, and sex were included in exploratory adjusted analyses, breed and other clinical variables such as treatment status, disease severity, disease subtype, diuretic therapy, and fluid balance were not incorporated into multivariable models because of limited sample size, sparse breed categories, and incomplete standardization. Factors such as diuretic therapy, disease severity, and fluid balance may influence hematologic and biochemical parameters and should be considered as potential confounders.

The sample size, particularly within subgroup analyses, was relatively limited, which may affect statistical power and the generalizability of the findings. Future prospective studies with standardized clinical characterization and longitudinal follow-up are warranted to further elucidate the mechanisms of iron dysregulation in canine cardiac disease.

Finally, this study included dogs with naturally occurring cardiac disease and therefore reflects the clinical heterogeneity encountered in routine practice, including differences in underlying cardiac diagnosis, disease stage, treatment status, and comorbidities. This heterogeneity is particularly relevant for the CCD group because of its small sample size. Accordingly, the present findings should be interpreted as exploratory and hypothesis-generating rather than as disease-specific diagnostic criteria. Future studies should evaluate iron metabolism in larger and more homogeneous cohorts, particularly dogs with MMVD stratified by ACVIM stage and treatment status.

### 4.8. Clinical Relevance and Future Directions

Interpretation of iron homeostasis in dogs remains challenging, as disturbances in iron metabolism are still largely considered in the context of overt anemia, and comprehensive assessment of iron regulation outside this framework is uncommon in veterinary medicine. The present study suggests that iron handling may already be altered in dogs with cardiac disease before clinically apparent anemia develops. Therefore, these findings support the concept that iron status, inflammatory activity, and nutritional–metabolic markers may be relevant earlier in the disease course than is currently recognized.

From a clinical perspective, the substantial overlap between groups indicates that the evaluated biomarkers should not be interpreted as standalone diagnostic tests or used in isolation to guide therapeutic decisions in individual dogs. This is not unexpected, as assessment of iron status is complex, particularly in inflammatory disease. In human medicine, serum iron, ferritin, transferrin saturation, and inflammatory markers are interpreted together, because ferritin may remain normal or increased during inflammation despite reduced iron availability, while serum iron concentrations may vary according to inflammatory status, disease severity, and systemic metabolic demands. Similarly, in dogs with cardiac disease, a normal ferritin concentration should not necessarily be interpreted as evidence of normal iron availability, and serum iron values should be considered in the context of CRP, albumin, TIBC/UIBC, hematologic indices, and clinical status.

The aim of the present study was not to establish diagnostic cutoffs for iron deficiency or to propose treatment thresholds, but rather to characterize group-level alterations in iron handling in dogs with cardiac disease. While exploratory and limited by sample size, the findings indicate that reduced circulating iron and altered iron-binding capacity can occur even in the absence of overt anemia, showing similarities to patterns described in human heart failure, where inflammation and altered iron handling are increasingly recognized. However, because this study was not designed to evaluate the effect of individual clinical variables on iron metabolism, factors such as treatment status, disease severity, diet, and comorbidities could not be fully assessed analytically. Where available, these clinical variables were therefore summarized descriptively to provide context for interpretation.

Larger longitudinal studies incorporating dietary assessment, reticulocyte indices, transferrin saturation, and functional markers of erythropoiesis are warranted to clarify temporal relationships between inflammation, nutrient redistribution, and iron availability. Future studies should also determine whether disease-specific reference limits or clinically useful cutoffs, as used in some human inflammatory and cardiac conditions, can be established for dogs, and whether integrated iron-related biomarker profiles are associated with outcome, exercise tolerance, quality of life, or response to iron-directed interventions.

## 5. Conclusions

Dogs with naturally occurring cardiac disease, particularly those presenting with ADCHF, show reduced circulating iron availability and alterations in iron-related biomarkers, suggesting that disruption of iron homeostasis may occur in canine cardiac disease.

The inflammatory state and the iron-related alterations are consistent with reduced iron availability, possibly identifying cardiac disease as a condition in which nutrient–immune interactions become clinically relevant.

## Figures and Tables

**Table 1:** Selected clinical, hematologic and echocardiographic parameters of dogs from all three groups. ^1^ Acute decompensated congestive heart failure (ADCHF); ^2^ compensated cardiac disease (CCD); ^3^ 25% percentile (Q1); ^4^ 75% percentile (Q3); ^5^ left-atrium-to-aorta ratio (La/Ao); ^6^ standard deviation (SD); ^7^ left ventricular internal dimension in diastole normalized (LVVDn); ^8^ C-reactive protein (CRP); ^9^ creatinine (CREA); ^10^ total protein (TP); ^11^ albumin (ALB); ^12^ alanine aminotransferase (ALT).

Parameter	Unit Reported	Group			Reference Interval
		ADCHF ^1^	CCD ^2^	Healthy	-
Weight kg	median (Q1–Q3) ^3,4^	5.0 (3.0–6.7)	12.0 (6.2–25.5)	16.0 (7.7–21.0)	-
Age years old	median (Q1–Q3)	12.0 (10.5–13.0)	14.0 (10.0–15.0)	7.0 (3.0–10.0)	
La/Ao ^5^	mean (±SD) ^6^	1.97 (±0.47)	1.37 (±0.25)	-	<1.6
LVDDn ^7^	mean (±SD)	1.98 (±0.48)	1.43 (±0.24)	-	<1.7
HCT %	mean (±SD)	44.5 (±6.45)	44.8 (±5.9)	50.3 (±5.37)	37–55%
RBC 10^6^/µL	mean (±SD)	6.5 (±0.95)	6.5 (±1.07)	7.2 (±0.77)	5.5–8.5 × 10^6^/µL
RDW %	median (Q1–Q3)	12.9 (12.3–13.6)	12.2 (11.7–12.9)	12.0 (11.8–12.6)	12–15%
CRP mg/L ^8^	median (Q1–Q3)	24.5 (6.72–60)	19.0 (3.27–67.65)	1.3 (0.5–2.4)	<10 mg/L
CREA mg/dL ^9^	median (Q1–Q3)	0.9 (0.8–1.3)	0.8 (0.6–1.0)	1.0 (0.8–1.1)	0.5–1.5 mg/dL
TP g/dL ^10^	median (Q1–Q3)	6.0 (5.4–7.0)	6.3 (6.0–6.8)	6.1 (5.8–6.2)	5.5–7.5 g/dL
ALB g/dL ^11^	mean (±SD)	3.4 (±0.47)	3.35 (±0.34)	3.61 (±0.24)	2.6–4.0 g/dL
ALT U/L ^12^	median (Q1–Q3)	68.0 (42.0–106.5)	49.0 (34.0–150.5)	42.0 (27.0–59.5)	10–100 U/L
Ascites	number of cases	N = 3 (12%)	N = 0	N = 0	-
Pleural effusion	number of cases	N = 1 (4%)	N = 0	N = 0	-
Diuretics listed among cardiac medication	number of cases	N = 25 (100%)	N = 4 (29%)	N = 0	-

## Data Availability

The datasets generated and analyzed during the current study consist of clinical data from client-owned animal patients. Due to data protection and client confidentiality considerations, the full dataset is not publicly available. Anonymized data supporting the findings of this study are available from the corresponding author upon reasonable request and with appropriate institutional approval. All relevant data necessary to interpret the results are included within the article. Data were made available to the reviewers during peer review.

## Abbreviations

The following abbreviations are used in this manuscript:

TIBC	Total iron-binding capacity
UIBC	Unsaturated iron-binding capacity
ADCHF	Acute decompensated congestive heart failure
CCD	Compensated cardiac disease
MMVD	Myxomatous mitral valve disease
ID	Iron deficiency
FID	Functional iron deficiency
TSAT	Transferrin saturation
ACVIM	American College of Veterinary Internal Medicine
RDW	Red cell distribution width
CRP	C-reactive protein
